# Review of methods used by chiropractors to determine the site for applying manipulation

**DOI:** 10.1186/2045-709X-21-36

**Published:** 2013-10-21

**Authors:** John J Triano, Brian Budgell, Angela Bagnulo, Benjamin Roffey, Thomas Bergmann, Robert Cooperstein, Brian Gleberzon, Christopher Good, Jacquelyn Perron, Rodger Tepe

**Affiliations:** 1 Canadian Memorial Chiropractic College, 6100 Leslie St., Toronto, Ontario, Canada; 2 Private Practice of Chiropractic, Burlington, Canada; 3 Private Practice of Chiropractic, Toronto, Canada; 4 Private Practice of Chiropractic, Calgary, Canada; 5 Northwestern Health Sciences University, Bloomington, MN, USA; 6 Palmer College of Chiropractic, San Jose, CA, USA; 7 University of Bridgeport College of Chiropractic, Bridgeport, CT, USA; 8 Logan College of Chiropractic, Chesterfield, MO, USA

**Keywords:** Diagnostic accuracy, Validity, Reliability, Spinal manipulation, Chiropractic

## Abstract

**Background:**

With the development of increasing evidence for the use of manipulation in the management of musculoskeletal conditions, there is growing interest in identifying the appropriate indications for care. Recently, attempts have been made to develop clinical prediction rules, however the validity of these clinical prediction rules remains unclear and their impact on care delivery has yet to be established. The current study was designed to evaluate the literature on the validity and reliability of the more common methods used by doctors of chiropractic to inform the choice of the site at which to apply spinal manipulation.

**Methods:**

Structured searches were conducted in Medline, PubMed, CINAHL and ICL, supported by hand searches of archives, to identify studies of the diagnostic reliability and validity of common methods used to identify the site of treatment application. To be included, studies were to present original data from studies of human subjects and be designed to address the region or location of care delivery. Only English language manuscripts from peer-reviewed journals were included. The quality of evidence was ranked using QUADAS for validity and QAREL for reliability, as appropriate. Data were extracted and synthesized, and were evaluated in terms of strength of evidence and the degree to which the evidence was favourable for clinical use of the method under investigation.

**Results:**

A total of 2594 titles were screened from which 201 articles met all inclusion criteria. The spectrum of manuscript quality was quite broad, as was the degree to which the evidence favoured clinical application of the diagnostic methods reviewed. The most convincing favourable evidence was for methods which confirmed or provoked pain at a specific spinal segmental level or region. There was also high quality evidence supporting the use, with limitations, of static and motion palpation, and measures of leg length inequality. Evidence of mixed quality supported the use, with limitations, of postural evaluation. The evidence was unclear on the applicability of measures of stiffness and the use of spinal x-rays. The evidence was of mixed quality, but unfavourable for the use of manual muscle testing, skin conductance, surface electromyography and skin temperature measurement.

**Conclusions:**

A considerable range of methods is in use for determining where in the spine to administer spinal manipulation. The currently published evidence falls across a spectrum ranging from strongly favourable to strongly unfavourable in regard to using these methods. In general, the stronger and more favourable evidence is for those procedures which take a direct measure of the presumptive site of care– methods involving pain provocation upon palpation or localized tissue examination. Procedures which involve some indirect assessment for identifying the manipulable lesion of the spine–such as skin conductance or thermography–tend not to be supported by the available evidence.

## Background

The primary focus of chiropractic practice is the evaluation and management of disorders of the neuromusculoskeletal system. Treatment of these disorders includes manual manipulation procedures directed toward normalizing alterations of the locomotor system [1]. With increasing evidence of clinical benefit for spinal manipulation (for example, see Gross et al. [2]; Rubinstein et al. [3]; Bronfort et al. [4]), there is growing interest in identifying the appropriate indications for localizing the site of care.

Patient evaluation can be viewed as a sequence of procedures designed to progressively narrow the focus of attention, first to region then local site and, sometimes, tissue. In the ideal circumstance, the clinical value of a test or maneuver is based more on the health consequences from using it rather than on its accuracy alone [5]. In the case of the spine, however, over 90% of complaints have been categorized as a heterogeneous grouping which might be termed “non-specific” spinal pain [6]. An additional 5% to 9% are attributed to neurological deficits referable to the spine. Currently, there is no consensus on the mechanism(s) or identity of the pain generators of non-specific spinal pain. Even the validity and impact on care delivery of recent clinical prediction rules remain uncertain [7]. Consequently, there is no gold standard for diagnosis, in the traditional sense. A more pragmatic concept, the clinically important manipulable or functional spinal lesion, has emerged that recognizes the heterogeneous clinical manifestations from local tissue strains and altered neuromotor control [8,9] seen in these patients.

As in other fields of medicine, it is the clinical presentation, rather than a gold standard diagnosis, that directs decision making [10-12]. Faced with the dilemma of an absent gold standard [10] and yet a duty to care [13], one of the options is to evaluate the consequences of the disorder being treated. For example, there currently is no means to differentiate the specific pain generator in the presence of multiple abnormalities on imaging [14]. For a patient who has radicular pain, an imaging abnormality that has the potential to produce symptoms must be concordant with the location and distribution of signs and symptoms to correctly frame the diagnosis. This is the framing of gold standard that is adopted for use within the remainder of this article.

The pathophysiologic consequences of manipulable lesions have been loosely aggregated, primarily from common clinical wisdom and collective experience, into related categories; Pain, Asymmetry, relative Range of motion, changes in Tissue temperature/texture/tone, and findings from Special tests (P.A.R.T.S.). Thus, P.A.R.T.S. is commonly viewed [15] as a required foundation for manipulation treatment and is likely the most widely utilized method to justify a treatment application site [16]. There appear to be six core and overlapping constructs underlying the P.A.R.T.S. These constructs define somatotopic relationships between the patient’s signs and symptoms and biological substrates including dermatomes, sclerotomes and myotomes, as detailed below:

•Pain–P.

○ Findings from self-report and the reproduction of pain through diagnostic manoeuvres are spatially related to the local presence of pathology/dysfunction.

•Asymmetry–A.

○ Anatomical landmarks present an observable cephalo-caudal pattern, within the sagittal plane, and/or a bilateral symmetry in their location, motion and compliance/stiffness in response to challenge or perturbation.

•Range of motion–R.

○ Joints, within a linkage system, contribute a predictable proportion and path to the regional movement expressed by the linkage system as a function of task.

•Tissue temperature, texture, and tone–T.

○ Muscle, as both a sense organ and actuator, responds to pathology that is spatially related with hypertonicity, hypotonicity, hypertrophy or atrophy as a function of the primary tissue disease process present.

○ In the presence of pathology/dysfunction, a spatially consistent change in the relative ratio of fluid (edema) to cellular and acellular components is observable.

•Special tests–S.

○ In the presence of pathology/dysfunction, there is a spatially consistent neurogenic activity that demonstrates a muscular, kinematic, vascular, or secretory response that is observable.

When a course of manipulation is elected, the provider must use her/his clinical judgment, often employing the constructs above, to determine which procedures to apply and where. It is this topic that motivates the present work. To be clear, the topic of investigation was not to determine the bases for a judgement to apply manipulation, rather that once such a judgement has been made, what are the foundations to determine the site to which treatment would be directed. The purpose of this study is to identify the best available evidence as to what methods of assessment can inform the provider as to localization of treatment.

## Methods

The core question on where to site the application of manipulation treatment, while simple in concept, is remarkably complex to answer. The literature involves a breadth of evidence which often has been studied using heterogeneous methods or measures and requires contextual interpretation. The approach used was a hybrid method allowing for both a consensus process, after the work of Bigos et al. [17], Haldeman, Chapman-Smith and Petersen [18] and the RAND expert panels on the appropriateness in use of manipulation [19,20], with the use of explicit and systematic tools to evaluate the quality of evidence in the manner of a systematic review [21]. Unlike the evaluation of outcome-related studies that lend themselves to use of PRISMA [21], MOOSE [22] or RAMSES [23] guidelines, studies that evaluate the “accuracy” of tests require different criteria to appropriately assess the quality of studies and the potential for bias [24].

A team of reviewers was assembled under sponsorship of the Association of Chiropractic Colleges (ACC) in conjunction with the Council on Chiropractic Guidelines and Practice Parameters (CCGPP). Two team leads (JT & BB) were identified who together held prior experience and publications in methods of literature synthesis and/or analysis. Administrative support was provided by two recent graduate clinicians (AB & BR) who tracked article reviews and team member participation. A request for nominations to the review panel was sent to constituent members of the ACC and CCGPP. The panel of reviewers consisted of 7 members (1 PhD; 2 DC, PhD; 1 DC, MSc; 3 DC) representing 5 different chiropractic institutions. One reviewer was based in private practice, 5 DCs were in part-time practice or had recently retired from full time practice. The panel conducted reviews and developed consensus for conclusions presented in this report, independently from the sponsors.

Electronic searches were conducted using EBSCO host search engine within Medline, PubMed, CINAHL and ICL through July 2010. A series of terms were searched individually and concatenated with anatomical region (i.e. spine, vertebr*, cervical spine or neck, thoracic spine or mid back, lumbar spine or low back, sacroiliac joint or pelvis) and/or treatment type (i.e. manip* or mobilis* or mobiliz*) and/or discipline (e.g. chiropract*). The terms list was inclusive of the following: applied kinesiology, arm fossa test, current perception threshold, diagnos*/ assessment, electromyography/electromyograph*/emg, joint play/challenge, leg length inequality/asymmetry, muscle testing/manual muscle test*, neurocalometer/neurocalomet*, orthopedic/orthopedic test, pain/pain provocation, palpation–static and motion, PARTS (pain, asymmetry, range of motion, tone and texture and special tests)’ , physical examination, posture, radiography/radiograph*/x-ray, range of motion/rom.

Hand searches of publication references and of archives were also conducted. References retained for review by the team had to meet five prospective inclusion criteria: 1) topics focused on diagnostic validity and/or reliability for methods of patient evaluation used to identify the site of care by manual treatment, 2) articles were primary source research reports containing original data obtained from humans, 3) examiners were experienced providers or health sciences students, 4) for validity studies, at least some of the subjects must have been symptomatic or have had a known anatomical anomaly, and 5) publication was in the indexed and peer reviewed English language literature. No limits were placed on the date of publication.

In addition to searching for studies pertaining to individual assessment procedures, studies that investigated clusters of tests were also retrieved. Search topics were informed by the background focus on spinal manipulation and the P.A.R.T.S. constructs. Searching was assisted and full text copies of papers were made available for administrative review through the services of the Cleveland Chiropractic College and Canadian Memorial Chiropractic College Libraries.

The administrative screening confirmed eligibility of the paper according to the prescribed inclusion criteria checklist. Subsequently, each eligible paper was assigned to one of two research assistants who worked independently of the reviewers and who extracted data which was placed in an evidence table. In addition to manuscript metadata (author, journal etc.), the evidence table recorded the spinal region investigated, the reference standard (‘gold standard’), disease spectrum of patients/subjects, whether the manuscript referred to intra-rater reliability, inter-rater reliability or validity, a summary of the experimental design, the statistics/outcome measures reported, and any limitations to be taken into consideration in interpreting the findings.

Each article then was assigned, on a rotating basis, to two reviewers. Within the two classes of studies, validity and reliability, papers were scored independently by each reviewer based on their content using the QUADAS [25,26] and QAREL [27] instruments, respectively. Some validity studies contained nested reliability studies for specific aspects of the methods under investigation. These minor reliability exercises were not scored independently. Each member of the team participated in training with respect to use of the two instruments under supervision of an experienced user (BB). Exercises consisting of review and scoring of validity and reliability studies were conducted using literature unrelated to the purpose of this study. Scored checklists were returned to the research assistants, who were not part of the review team, and results were recorded. If there was disagreement between reviewers on any element of the QUADAS or QAREL ratings, communication over the point of disagreement was facilitated by the research assistant to attempt reconciliation. For any disagreement not reconciled between reviewers, the topic was set aside for the full review panel meeting and discussion.

Item response theory was used to weight QUADAS and QAREL items based on the inverse of their prevalence among the papers reviewed (see Nakayama and Budgell 2009) [28]. A separate manuscript, under preparation, describes the validation of this approach for QUADAS and QAREL. In brief, however, papers were not simply awarded 1 point for each criterion which they satisfied. Rather, criteria were weighted and their ability to discriminate between papers of higher and lower quality was confirmed. These scores only indicate relative quality within the cohort of papers reviewed and only based on the QUADAS and QAREL checklists. The full listing of scores by primary author and date may be found in Tables 1 and 2. Question level data for each paper scored with QUADAS and QAREL may be found in Additional files 1 and 2, respectively.

**Table 1:** Rankings of validity studies per QUADAS scores

**Article ID**	**Percentile**	**Rank**	**Article ID**	**Percentile**	**Rank**
Abbott_2003	78	19	King_2007	77	20
Abbott_2005	92	6	Knutson_2002	77	21
Abbott_2006	100	1	Landel_2008	90	8
Beattie_1990	83	14	Laslet_2003	73	28
Bierma-Zeinstra_2001	90	8	Laslet_2005	94	2
Brismee_2006	41	40	Leach_1993	91	7
Bryner_1994	64	31	Lebeouf_1990	75	26
Caruso_2000	27	44	Leboeuf-Yde_2000	93	3
Chafetz_1988	76	24	Leboeuf-Yde_2002	100	1
Chakraverty_2007	85	10	Lehman_2002	77	23
Cibulka 1999	35	41	Levangie_1999	92	5
Cooperstein_2003	59	35	McCulloch_1993	100	1
Cooperstein_2004	84	11	Montgomery_1995	55	37
Dankaerts_2006	78	18	Nansel_1988	64	32
Diakow_1988	48	38	Ogince_2007	78	17
Erikson_1996	32	43	Osterbauer_1996	33	42
Fernandez-de-las-Pinas _2005	57	36	Peterson_2008	77	22
Fortin_1997	45	39	Petrone_2003	93	4
Fritz_2011	83	12	Phillips_1996	100	1
Fryer_2010	69	29	Pollard_2006	84	11
Haas&Peterson_1992	75	25	Roy_2008	87	9
Harlick_2007	79	16	Sandmark_1995	90	8
Harrison_1998	64	33	Taylor_1990	83	13
Harrison_2003	92	5	Viitanen_2000	83	12
Harrison_2004	62	34	Yamashita_2002	67	30
Humphreys_2004	84	11	Zaproudina_2006	74	27
Imoto_2007	83	15			
Jende_1997	92	6			
Jull_1988	100	1			

Summary scores (not shown) were generated using weightings of QUADAS checklist items, where weights of individual items were based on the inverse of their prevalence within this cohort of articles. Subsequently, articles were ranked according to their normalized (Percentile) score and their relative position (Rank) within this cohort of articles.

**Table 2:** Rankings of reliability studies per QAREL scores

**Article ID**	**Percentile**	**Rank**	**Article ID**	**Percentile**	**Rank**	**Article ID**	**Percentile**	**Rank**
Agarwal_2005	26	73	Danneels_2001	18	82	Herzog_1989	37	56
Amiri_2003	24	79	Degenhardt_2005	30	65	Hicks_2003	60	31
Antos_1990	38	53	Degenhardt_2010	66	16	Hinson_1998	22	80
Arab_2009	62	26	Diakow_1988	17	83	Holmgren_2008	85	6
Bergstrom_1986	11	87	Downey_2003	77	9	Holt_2009	32	60
Bertilson_2003	61	28	Fjelhner_1999	69	13	Hoppenbrouwer_2006	48	41
Binkley_1995	47	43	Fortin 1997	13	86	Horneij_2002	46	46
Bo_1997	29	67	French_2000	41	50	Hsieh_1990	16	85
Bockenhauer_2007	22	80	Fryer_2005	78	8	Hubka_1994	33	59
Boline_1988	41	49	Fryer_2006	53	37	Hungerford_2007	32	61
Boline_1993	38	53	Fuhr_1989	16	85	Hunt_2001	22	80
Breum_1995	32	60	Gemmell_1990	46	47	Jackson_1993	64	19
Brismee_2005	46	47	Ghoukssian_2001	17	84	Jackson_1998	26	70
Brismee_2006	64	18	Gibbons_2002	26	72	Jende_1997	40	51
Byfield_1992	29	67	Gross_1998	24	76	Johansson_2006	77	9
Calderon_1994	38	55	Haas_1990	37	57	Keating_1990	61	28
Carmichael_1987	39	52	Haas_1992	72	11	Kilpikoski_2002	46	47
Chakraverty_2007	30	66	Haas_1993	100	1	Kim_2007	22	80
Chiarello_1993	32	61	Haas_1995	100	1	Kmita_2008	100	1
Christensen_2002	77	9	Hall_2004	61	30	Kokmeyer_2002	63	21
Cibulka_1999	24	78	Hanada_2001	24	77	Laslett_1994	46	47
Clare_2004	53	36	Hanten_2002	35	58	Latimer_1998	10	88
Clare_2005	31	64	Harrison_2003	26	73	Leach_2003	69	12
Cleland_2006	46	47	Hart_2007	53	39	Leard_2009	63	23
Comeaux_2001	61	30	Haswell_2004	68	14	Lee_2002	31	64
Cook_2004	37	56	Hawk_1999	29	67	Love_1987	68	15
Cooperstein_2010	61	27	Haynes_2002	22	80	Ludtke_2001	47	45
Cowherd_1992	0	91	Heiderscheit_2008	22	80	Lundberg_1999	46	47
Croft_1994	68	14	Heiss_2004	37	56	Maher_1994	46	47
**Article ID**	**Percentile**	**Rank**	**Article ID**	**Percentile**	**Rank**	**Article ID**	**Percentile**	**Rank**
Maigne_2009	76	10	Plaugher_1991 #67	62	24	Vikai_Juntura_1987	63	22
Marcotte_2002	29	67	Plaugher_1993	22	80	Vincent-Smith_1999	32	60
Marcotte_2005	6	89	Pool_2004	46	47	Weiner_2006	24	78
Mayer_2004	20	81	Potter_2006	61	29	Woodfield_2011	46	47
McCombe_1989	31	64	Qvistgraad_2007	92	4	Younquist_1989	84	7
McKenzie_1997	53	38	Razmjou_2000	46	47			
McPartland_1996	46	47	Rhodes_1995	54	34			
Meijne_1999	32	63	Rhudy_1988	46	47			
Mior_1985	48	40	Riddle_2002	31	64			
Mootz_1989	32	62	Robinson_2007	94	3			
Moran_2001	48	42	Robinson_2009	54	35			
Nansel_1989	48	42	Rouwmaat_1998	92	5			
Nguyen_1999	62	26	Roy_2006	18	82			
Normand_2007	22	80	Schneider_2007	25	74			
O'Haire_2000	38	54	Schneider_2008	77	9			
Olson_1998	24	79	Seay_2007	28	68			
Olson_2009	42	48	Smedmark_2000	46	47			
Owens_2000	17	83	Solinger_2000	24	75			
Owens_2004	27	69	Strender_1997 (160)	46	47			
Owens_2007	26	71	Strender_1997 (167)	38	53			
Paatelma_2010	38	53	Sweat_1988	5	90			
Paulet_2009	47	44	Tong_2006	30	66			
Paydar_1994	26	72	Tousignant_2001	29	67			
Peterson_2004	55	33	Toussaint_1999	65	17			
Petrone_2003	37	56	Troke_1998	62	25			
Phillips_1986	63	20	Troke_2007	55	32			
Piva_2003	46	47	Troyanovich_1999	94	2			
Piva_2006	94	2	Van Dillen_1998	40	51			
Plaugher_1991 #107	100	1	VanSuijlekoma_2000	24	78			

Summary scores (not shown) were generated using weightings of QAREL checklist items, where weights of individual items were based on the inverse of their prevalence within this cohort of articles. Subsequently, articles were ranked according to their normalized (Percentile) score and their relative position (Rank) within this cohort of articles.

The full team met to review scores and to summarize the evidence. Seed definitions for quality of evidence, prepared in advance of the team meeting by the project support staff, were derived from the earlier work by Bronfort et al. [4]. Final definitions for consensus were adopted by vote at the outset of the meeting. Each reviewer presented summary overviews on up to 3 of the patient assessment methods, discussing the quality and content of each paper and the overall quality and content of the body of literature on the topic. Discussion was conducted following presentation of each summary, using a facilitated round-robin method [29,30]. Thus, after each reviewer had made their presentation, each participant was addressed, in turn, with the opportunity to comment with respect to a topic and to ask questions until he/she was satisfied. Following one round, a second round was conducted where each member was offered a further opportunity to emphasize or introduce comments. Summary notes on comments were maintained electronically and on a white-board. Then, by consensus vote, the group ranked the quality of the body of literature on each topic and the degree to which the literature supported the use of the modality in question. Consensus summary scores for each paper served to rank the quality of each article as a percentile within its cohort of reliability or validity papers. Where both intra- and inter-examiner reliability was assessed, we chose to weight only the latter as it speaks more to the generalizability of use. Similarly, where papers reviewing literature on reliability and/or validity were used to establish topic background and context, the precursor studies were not independently rated.

A mechanism for a minority opinion report was prospectively designed for the circumstance where a consensus by simple majority could not be achieved. Final recommendations on whether or not the diagnostic tool should be utilized in practice came from the consensus rankings.

## Results

The interprofessional relevance of the literature retrieved will be immediately evident. Not unexpectedly, while the search strategies were narrowed to those topics arising from or related to the PARTS concepts and the discipline focus, work from authors of various disciplines were represented and suggests that the findings reported herein have broader application than to the chiropractic discipline alone.

**Figure 1:**
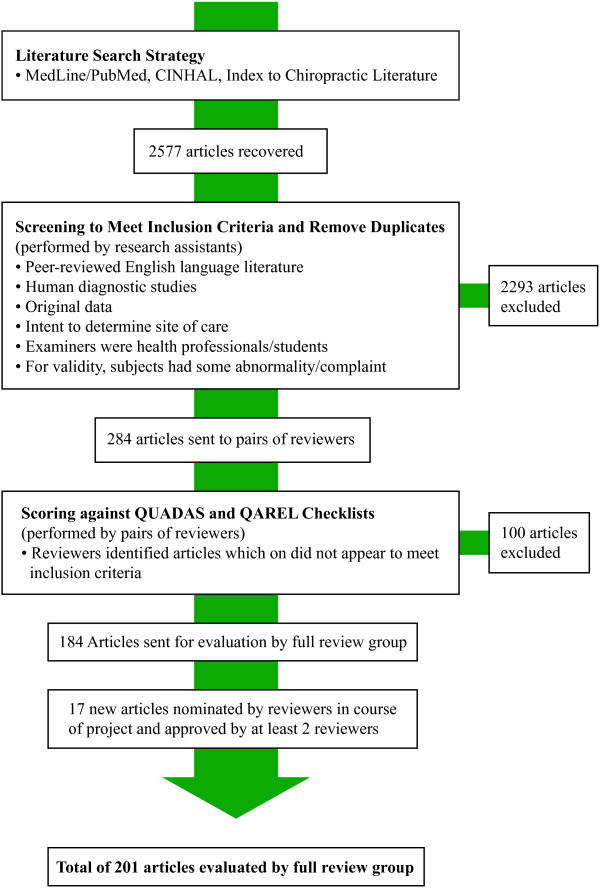
Literature search strategy.

A total of 2577 titles identified through the electronic literature search were screened, and, from these, 184 articles met all inclusion criteria and were accepted into this study. In the course of the project, an additional 17 articles were brought forward by reviewers, determined to meet the inclusion criteria and were incorporated into the analysis. Hence, the great majority of studies identified in our initial literature search were excluded from analysis. The most common reasons were that the studies were not directed towards identifying the site of care, region or segment, to be treated, and that the study involved subjects who were, in the main or entirely, healthy (Figure 1). Topic areas were aggregated following the P.A.R.T.S. constructs with the number of studies at each level of evidence listed with the section title. Some papers were included in more than one section if they investigated more than one assessment modality. A summary of the literature representative of the breadth of quality scores is provided under each heading with panel recommendations and ratings of the evidence. No conflicts on QUADAS or QAREL scores remained between reviewer pairs for the consensus meeting agenda. Similarly, no minority reports resulted from the consensus rankings.

### Consensus and levels of evidence

Consensus was defined as an agreement of greater than or equal to 70% by panel vote. Following the reviewer ranking according to QUADAS and/or QAREL scores, each paper was considered as providing a ‘high’ level of evidence if it achieved a quality score ≥ 70%, a ‘moderate’ level of evidence if its score ranged between 40% and 69%, and a “low” level of evidence if the score was ≤ 39% (see Table 3, Column 2). These criteria were set based on what appeared, early in the study, to be natural clusterings of scores.

**Table 3:** Levels of evidence defined by quality scores of individual papers

**Level**	**Quality scores for**	**Quality definition for**
	**individual papers**	**topic/procedure**
High	≥ 70%	Two high or one high AND two moderate scores
Moderate	≥ 40%	One high OR two moderate scores
	< 70%	
Low	< 40%	Everything else

Interpretations of reliability Kappa (ĸ) scores follow the work of Viera and Garrett [31] and for intra-class correlations (ICC) that of Fleiss [32]. Definitions relating score value with levels of agreement are provided, for convenience, in Table 4.

**Table 4:** Definitions of levels of agreement for reliability study scores

**Kappa**		**Intra-class correlation**	
Range	Definition	Range	Definition
< 0	Less than chance	< 0.4	Poor
0.01–0.20	Slight	0.4-0.75	Moderate
0.21– 0.40	Moderate	0.75-0.9	Good
0.61–0.80	Substantial	> 0.9	Excellent
0.81–0.99	Near perfect		

A summary of recommendations, themselves reported at the end of each section below, can be found in Tables 5 and 6. Table 5 provides a description of each of the five possible types of recommendation while Table 6 gives a summary of recommendations for each category of evaluation method. It should be emphasized for clarity that there were a significant number of the studies that offered evidence on more than one assessment approach. The definitions for rating of evidence (Table 3) were based on the number of papers for which the QUADAS or QAREL scoring met or exceeded the threshold level.

**Table 5:** Definitions for each of the possible types of recommendation

**Recommendation**	**Description**
Favourable	Favourable for general use by clinicians to determine site of care
Favourable with limitations	Favourable for determining site of care although limits exist such as number and quality of studies, limited generalizability, etc.
Unclear	Based on the evidence available, it is unclear whether or not this procedure should be recommended for use
Unfavourable with exceptions	Procedure is not recommended for general use but may be used in limited circumstances (e.g. other techniques unavailable.)
Unfavourable	Procedure is not recommended for use (limited number of studies, significant flaws in methods, not generalizable, high quality evidence against validity and/or reliability)

**Table 6:** Summary of recommendations for each category of evaluation method

**Evaluation method**		**Summary**	**Quality of**	**Recommendation**
			**evidence**	
Complaint History		Gives context to the complaint and increases the reliability of the interpretation of physical findings.	Moderate	Favourable
Pain provocation				
	Tenderness	Localizes region/tissues of involvement	High	Favourable
	Orthopedic manoeuver	Pain with movement localizes region/tissue of involvement	High	Favourable
Asymmetry				
	Posture	Antalgia, kyphosis, lordosis, scoliosis	High	Favourable
		Localizing to site of care	High	Unfavourable
Stiffness	Palpatory manual	Passive physiologic/accessory motion, joint springing, overpressure testing	High	Unclear
	Instrumented	Questions of generalizability	Low	Favourable with limitations
Palpation	Static	Identifying major anatomical landmarks	High	Favourable with limitations
		Localizing to site of care	High	Unclear
	Motion	Enhanced if pain provocation present	High	Favourable with limitations
Leg Length Inequality		Pelvic assessment; method dependent	High	Favourable with limitations
Manual Muscle Testing		Strength grading to localize root involvement.	Moderate	Favourable
		Non-pathologic altered function	Moderate	Unfavourable
Range of motion		Localization to region	High	Favourable
Tissue temperature, texture, tone		Thermography/thermometry of the lower limb in confirming frank sciatica	High	Favourable
		Paraspinal skin temperature to locate site of care	High	Unfavourable
		Texture-skin rolling	Moderate	Favourable
Specialized Tests	Current Perception Threshold	Frank neuropathy with sensory deficit	High	Favourable
	Galvanic skin response	Localizing to site of care	Moderate	Unfavourable
	Surface EMG	Flexion-relaxation phenomenon to target lumbar region	High	Favourable
		Localizing to site of care	Moderate	Unfavourable
	Radiographic imaging	Localizing to site of care	High	Unfavourable
Integrated P.A.R.T.S. Montages		Localizing to site of care beyond individual component contributions	Moderate	Unclear

### Pain

[Quality scores: Validity 0 low, 1 moderate, 11 high; Reliability 12 low, 17 moderate, 1 high].

Provocative manoeuvres, evoking relief or aggravation of familiar pain, augmented by historical factors, have been used to localize the suspected pain generator throughout the spine [33-67].

### Pain history

[Quality scores: Reliability 0 low, 5 moderate, 0 high].

In general, reliability of evaluations suffers unless pain provocation or pain history associated with the physical examination are taken into account [34-39]. In work on the cervical spine by Bertilson et al. [37], the awareness of complaint history enhanced reliability, measured by kappa, from 0.57 to 0.67 for evaluation of dermatomal sensitivity to pain, and from 0.4 to 0.49 for tenderness assessment. Cleland et al. [39] conducted a wide ranging study of reliability for history and physical findings related to neck pain. Categorical variables from the history that resulted in very good to excellent reliability (0.72 ≤ ĸ ≤ 1.0) included mode of onset, nature of symptoms, prior episodes, downward or upward looking, and sleeping postures aggravating symptoms. The inter-rater reliability of patient classification ranged from 0.68 ≤ ĸ ≤ 0.88, with judgments made strictly from recorded data on forms showing the lower reliability [38]. Fortin [35], in a low quality study, looked at the validity of patients pointing and thereby localizing symptoms to the sacroiliac joint. Sixteen subjects received provocative joint injections. All were positive for local pain. A subset of 10 subjects was further evaluated for comorbid discogenic or zygapophyseal joint pain generators with negative results for these conditions.

Recommendation: Favourable, based on moderate quality evidence, for use of pain history to increase reliability of symptom provoking findings during the assessment of site to apply treatment.

### Pain on provocation–tenderness

[Quality scores: Validity 0 low, 0 moderate, 5 high; Reliability 5 low, 7 moderate, 2 high].

The finding of tenderness connotes an unusual or increased sensitivity to pressure on palpation that localizes to the area examined. Where possible, the sensitivity of a site is evaluated by comparison to its contralateral asymptomatic counterpart. Twenty-two papers dealing with pain/tenderness on palpation were reviewed [33,40-50]. Bryner [43] used patient self-report of painful region as a reference standard to evaluate validity of examiner marked pain drawings for areas of tenderness to palpation. Sensitivity (0.73) and specificity (0.98) were excellent between the examiner and patient reports with respective positive (0.78) and negative (0.96) predictive values. Tenderness of the cervical articular pillars was evaluated in patients with neck pain and in healthy controls by Sandmark and Nissel 1995 [45]. High sensitivity (0.82) and specificity (0.79), with moderate positive (0.62) and high negative (0.91) predictive values were found. Leboeuf-Yde et al. [48], in a high quality study, looked at tenderness of the spinous processes on percussion, finding tenderness to have low sensitivity (0.39) and positive predictive (0.41) values with high specificity (0.78) and negative predictive (0.78) values. Lundberg and Gerdle [47] found substantial agreement between abnormal passive mobility tests of the lower lumbar spine and segmental pain provocation tests at L4/5 (ĸ=0.71) and L5/S1 (ĸ=0.67), somewhat better than the results reported by Hicks et al. (2003) [57]. Inter-rater reliability was substantial (ĸ=0.68) among seven examiners of neck pain patients in the report by Hubka and Phelan [44]. For the thoracic spinal region [50,51] and rib cage, substantial (0.62 ≤ ĸ ≤ 0.70) and almost perfect (ĸ=0.88) inter-rater reliability were reported for tenderness to pressure on the spinous and transverse processes, respectively. Tender points in the anterior and posterior soft tissue compartments of the upper neck [46] showed moderate inter-rater agreement (ĸ = 0.45).

King et al. [52] investigated the validity of palpation for cervical facet joint pain using response to medial branch nerve blockade as the reference standard. Whereas preliminary work by Jull et al. [53] reported 100% sensitivity and near 100% specificity, King et al. reported that manual palpation provided high sensitivities (0.88–0.89) and low specificities (0.39–0.50) but with low likelihood ratios of 1.4 to 1.8. The most common joint involvements in this study were C2-3 and C5-6. The high prevalence of C2-3 and C5-6 lesions, consistent with other reports, erodes the sensitivity values, drawing into question the ability to localize pain to the zygapophyseal joint itself [47].

The study by Viikari-Juntura et al. [33] evaluated inter-rater reliability of examination findings in 52 neck pain patients referred for myelography. Pain response to pin prick had moderate agreement (0.41 ≤ ĸ ≤ 0.51), with change in light touch (0.1 ≤ ĸ ≤ 0.6) and palpatory tenderness (0.24 ≤ ĸ ≤ 0.56) rating only fair to moderate. The inter-rater reliability of localizing pain to the upper, mid and lower cervical spine gave moderate (ĸ=0.53) agreement in the report by Maigne et al. [54]. A positive, but low, correlation (r=0.35, p<0.007) was noted between the number of areas of tenderness and the patient’s neck disability questionnaire scores. Agreement [54] for tenderness over neck muscle insertions ranged from fair (ĸ=0.33) to substantial (ĸ=0.62). Boline et al. [40,55] reported that pain on palpation over spinal/paraspinal osseous and soft tissue structures showed inter-rater reliability (0.48 < ĸ < 0.90) that was moderate to almost perfect in agreement.

Recommendation: Favourable, based on high quality evidence for validity and reliability of using tenderness to localize treatment.

### Pain provocation by orthopedic maneouvres

[Quality scores: Validity 1 low, 0 moderate, 2 high; Reliability 11 low, 15 moderate, 5 high].

In terms of the quality of the evidence, two studies [48,56] evaluating validity were of high quality, while one was of low quality [57]. Eleven reliability studies [41,57-66] were ranked as being of low quality, fifteen were ranked as moderate quality [33,34,36,37,39,67-76] and five, three of which contained nested reliability studies, were ranked as high quality [54,77-80].

Leboeuf et al. [48], using low back pain symptom history as a gold standard, evaluated the validity of provocation of pain by movement and seated forced extension with added manual pressure. Ranges of sensitivity (0.22–0.44), specificity (0.98–1.0), and positive (0.86–1.0) and negative (0.77–0.82) predictive values were found, depending on the direction of movement. Forced extension was sensitive at 0.78 with specificity at 0.71, a positive predictive value of 0.52 and a negative predictive value at 0.89. Hicks et al. [74] found moderate to near perfect reliability for the findings considered to be related to instability, including i) a painful arc in flexion (0.42 < ĸ < 0.77) pain on return to upright posture (0.42 < ĸ < 0.63) and the prone instability test (0.74 < ĸ < 1.00).

Van Dillen et al. [36], in a reliability study using 5 examiners, looked at symptom behavior during various postural tasks involving standing, sitting, supine and prone recumbency, hook lying and quadraped kneeling. For all 28 items, kappa values for inter-examiner reliability were ≥ 0.75 when symptom response was included, but reduced to ≥ 0.40 without them. Maher and Adams [34] found similar results when 3 pairs of examiners assessed spinal segment stiffness manually. Intra-class correlation coefficients were higher (0.67 ≤ ICC ≤ 0.72) when pain provocation was considered, but dropped dramatically (0.03 ≤ ICC ≤ 0.37) when it was not. Symptom provoking movement assessments, in the work of Haswell et al. [56], showed a hierarchy of reliability with movements which they termed side-bending (ĸ = 0.6), flexion-side-bend rotation (ĸ = 0.39), side-bend rotation (ĸ = 0.29), extension side-bend rotation (ĸ = 0.29) and rotation (ĸ = 0.17). Inter-rater reliability between two examiners was assessed by McCombe et al. [41] for straight leg raising, in two separate groups of low back pain patients using a correlation model. The angular position of pain onset and the maximum tolerated pain both correlated strongly (0.68 < r < 0.86). Maigne et al. [54] found good inter-examiner reliability for pain with cervical flexion (κ = 0.71) and extension (κ = 0.76).

A series of studies has examined the validity and inter-rater reliability of provocative postural manoeuvres to classify patients into categories of mechanical versus non-mechanical back pain.

Laslett et al. [79] used provocative discography as a gold standard to evaluate the classification of patients by “centralization” of pain during the examination. Sensitivity, specificity, and positive likelihood ratios for centralization were 40%, 94%, and 6.9 respectively. Clare et al. [81] studied the construct validity of the predicted response of extension in groups classified by provocative maneouvre as mechanical derangement versus non-derangement. The global perceived effect was significantly higher (p < 0.001) after treatment in the derangement group.

Inter-rater agreement for classification of low back patients into the subgroups of mechanical back pain, overall, has been good. Razmjou et al. [75] found very good agreement on derangement subsyndromes with κ = 0.96. Inter-rater agreement for presence of lateral shift, relevance of lateral shift, relevance of lateral component, and deformity in the sagittal plane were κ = 0.52, 0.85, 0.95, and 1.00, respectively. Kilpikoski et al. [76] found that agreement on centralization and direction of preference for helpful movements were substantial (0.7 < κ < 0.9). Clare et al. [82] found nearly perfect reliability for classifying both low back patients (κ = 0.89) and neck pain patients (κ = 0.84).

Laslett et al. [78] and Laslett et al. [79,80] used criterion validity to evaluate a cluster of orthopedic manoeuvres for pain which was considered to arise from the sacro-iliac joint, contrasting these manoeuvres against pain relief from intra-articular joint anesthesia. The choice of manoeuvres was based on earlier literature [67]; Kokmeyer et al. [69] citing “acceptable” inter-rater reliability for iliac distraction, thigh thrust, Gaenslen’s test, lateral recumbent iliac compression and prone sacral thrust. Using ROC curves they determined that, as a cluster, Gaenslen’s tests did not contribute to increased accuracy. With two or more of the remaining tests positive, the cluster sensitivity was reported at 0.94 with a specificity of 0.78 and predictive values positive at 0.68 and negative at 0.96.

Leboeuf-Yde et al. [48] evaluated the “stork” (a.k.a. Gillet) test for sacroiliac motion during the standing leg lift maneuver with generally poor results (sensitivity = 0.41, specificity = 0.75, positive predictive value = 0.39 and negative predictive value = 0.56). Levangie [56], in a high quality study, assessed the relationship between four clinical tests for sacroiliac function (Gillet, standing flexion test, sitting flexion test, and the supine-to-sit test) and both objectively measured pelvic torsion and low back pain. She found very little concordance with each other in any of the tests or with low back pain with the exception of the Gillet test (likelihood ratio 4.57). However all four tests had low to moderate sensitivity (range, 0.08 to 0.44) and moderate to high specificity (range, 0.64 to 0.93) with only moderate predictive values (range, 0.28 to 0.78).

The study by Viikari-Juntura et al. [33], rated a moderate quality study, evaluated inter-rater reliability of examination findings in 52 neck pain patients referred for myelography. Neck compression orthopedic testing varied in reliability (0.28 ≤ κ ≤ 0.77) according to head position at the time of axial compression. The reliability of brachial plexus tension tests was fair (κ = 0.35) while that of shoulder abduction relief was slight to fair (0.21 ≤ κ ≤ 0.40) and reliability of relief of symptoms by cervical traction was moderate (κ = 0.50). McCombe et al. [41] found provocation with movement to be variable in reliability depending on the direction of movement (0.1 ≤ κ ≤ 0.56).

Recommendation: Favourable, with high quality evidence for both validity and reliability in use of orthopedic manoeuvres to narrow the region of interest for applying treatment. Evidence supports seated forced extension; pain on lumbar motion (side-bending > flexion side-bending rotation > side-bend rotation > extension side-bend-rotation > rotation); three or more sacroiliac manoeuvres (iliac distraction, thigh thrust, lateral recumbent iliac compression and prone sacral thrust); cervical compression and traction tests; and McKenzie manoeuvres including lateral shift, relevance of lateral shift, relevance of lateral component, and deformity in the sagittal plane. A painful arc in flexion and/or on return to upright posture and the prone instability test may suggest local instability.

### Asymmetry

The assumptions of bilateral symmetry and some sort of structural and physiological axial pattern are common to a number of modes of patient examination. Absence of symmetry in some cases (e.g. scoliosis) is sufficient to result in a distinct diagnosis. In the majority of cases, however, it is the comparison from side-to-side or axially that is considered meaningful. In terms of assessment for localizing treatment to a specific site, the forms of examination involving symmetry include postural evaluation, palpation for stiffness of tissues/segments, static palpation of landmarks, segmental motion palpation, bilateral leg length measurement and manual muscle testing.

### Postural assessment

[Quality scores: Validity 0 low, 0 moderate, 1 high; Reliability 5 low, 4 moderate, 0 high].

Examiners inspect the relative positions of body segments/landmarks with respect to each other and with respect to an idealized configuration. Methods include visual inspection, photography, radiography and palpation of surface landmarks with or without aids. Deviations are noted and tend to direct further investigation. Nine articles which satisfied the inclusion criteria addressed postural assessment [39,41,48,68,83-87].

In the report by Lebouef-Yde et al. [48], validity of antalgic posture was assessed through the capture of data on subjects with known histories of back pain ranging from none in their life time to one or more episodes across preceding intervals up to one year. Antalgia was low in sensitivity (0.11) and positive predictive value (0.18) but high in specificity (0.80) and negative predictive value (0.69). Lordosis measurement, as the distance from the point of maximum thoracic kyphosis to maximum sacral kyphosis, was assessed by McCombe et al. [41]. Pearson correlations were calculated on measures taken by two examiners (0.67 < r < 0.7) from two sets of low back pain patients. Cleland et al. [39] found that the inter-rater reliability for evaluation of hyperkyphosis above T6 was better (0.69 < κ < 0.79) than for hypokyphosis (κ = 0.58). Photographic assessment of body surface landmarks [87] determined by palpation through tight fitting clothing resulted in poor reliability for judgment of kyphosis (κ = 0.441) and lordosis (κ = 0.327 ), but good reliability for scoliosis (κ = 0.769). In the work by Normand et al. [86], three examiners used palpation independently to locate surface landmarks and to place reflective markers. ICCs were calculated with both a conservative and liberal approach. Three landmarks from different angles served as input to a commercial computer program (PosturePrint®) which evaluated displacements of the head, rib cage, and pelvis (in degrees of rotation or millimeters of translation) from a normal upright stance. Depending on the vagaries of ICC analysis, results ranged from good to excellent (0.51 < ICC < 0.97).

Leard et al. [87] assessed the reliability of 22 clinical measures related to posture, including some manoeuvres that would be regarded as orthopedic tests. Intra-class correlations were reported for quantitative measures and weighted kappas for qualitative measures. Two clinicians, using a palpation meter (PALMeter™), produced ICCs ≥ 0.997 in measuring intra-rater reliability for sagittal pelvic inclination and ≥ 0.9661 for inter-rater reliability. Frontal plane inclination, however, had very low inter-rater reliability, with ICCs of approximately zero. Piva et al. [85] also looked at the iliac crest level in the frontal plane (i.e., pelvic obliquity) using a caliper mounted inclinometer. ICCs in their study were much higher than those of Leard et al. [87] 0.80 and 0.73 for standing and sitting, respectively.

French et al. [68] monitored the reliability of 5 examiners with respect to their conclusions as to the necessity to treat at a given site. A cluster of exam methods included postural inspection, patient self-report of pain characteristics, leg length assessment, motion and static palpation of the spine, as well as neurologic and orthopedic tests chosen at their discretion. Unfortunately, no breakdown of the analysis was reported by examination method. The yield in pooled reliability, for the cluster as a whole across the extent of the spinal column was only fair (κ = 0.27).

Cowherd et al. [83] attempted a criterion related validity study of common postural indices from digitized surface landmarks during erect stance using a triaxial, digitizing goniometer (Metrecom™). Results were compared to radiographs taken without changing position. While a stronger analysis would use an ICC, correlation of measures between the postural analysis and the radiographs showed only a weak association with significant (p<0.001) differences suggesting large offsets between the measures.

Recommendation: High quality evidence is favorable with limitations to the specificity of antalgia and reliability of postural assessment for kyphosis, lordosis and scoliosis. The evidence is unfavourable for the use of postural analysis to determine the local site of care.

### Palpatory stiffness

During the process of tissue palpation, the examiner attempts to assess the relative stiffness (conversely, compliance) to postural or applied load to a segment. Recent work in biomechanics has shown that the paraspinal soft tissues, particularly the multifidus muscles, differ in transverse stiffness based on patient posture and clinical state. Using the effective Young’s modulus, a direct measure of stiffness of the muscle estimated by force-deformation data from ultrasound elastography, Chan et al. [88] found a 50% to 300% increase (p<0.001) in elastic modulus depending on posture (prone, standing, 25° and 45° flexion). Similarly, with the exception of prone recumbency, chronic back pain patients showed a higher modulus than healthy subjects. The difference decreases with increased flexion, ranging from 30% to 14% in the mean. Effective spinal stiffness was similarly evaluated by Fritz et al. [89] before and after manipulation therapy. Improvements in self-reported disability correlated with decreases in stiffness (0.01≤ p ≤ 0.025). In parallel work [90], multifidus thickening was observed. Their results raise a question as to the interpretation of multifidus thickness, since 53.4% of subjects increased muscle dimension while 46.6% decreased after treatment.

The challenge that paraspinal stiffness poses to the clinician is finding evaluation methods that are able to discern these changes across the spectrum of disease and across the multiple spinal regions beyond the low back. Studies meeting the inclusion criteria fell into two categories, those that used manually applied forces and those using instrumentation.

### Stiffness- manual assessment

[Quality scores: Validity 0 low, 1 moderate, 3 high; Reliability 1 low, 8 moderate, 1 high].

Thirteen studies used manual assessment to evaluate segmental stiffness/mobility. The works of Abbott and Mercer [77], a high quality study, and Fernandez-de-las-Pinas et al. [91], a moderate quality study, both examined the validity of assessing hypomobility. Two high quality [42,92] studies and five moderate quality studies [34,39,93-95] and one low quality study [96] evaluated PA pressure or springing tests in the thoracic/lumbar spine, while evidence from two moderate quality studies [46,97] and one low quality study addressed the neck region [49].

Patients and healthy subjects were evaluated by four examiners using observation of active and abnormal ranges of motion, and motion palpation manoeuvres to assess for passive physiological intervertebral motion (PPIVM) and passive accessory intervertebral motion (PAIVM). Sensitivity ranged from 42% to 75%, while sensitivity was 35% to 89% [77]. Radiographic measures of segmental displacement on flexion/extension x-rays of the same subjects revealed that the number of segments falling more than 2 standard deviations below the mean of normal subjects is much higher in low back pain subjects than expected (χ2, p<0.001). Fernandez-de-las-Pinas et al. [91] evaluated manual lateral glide stiffness compared to intervertebral radiological motion in lateral bending. Radiologically visualized intervertebral motion on the hypomobile side (mean 19.1 mm, SD 2.1 mm) was 3.44 mm ± 1.9 mm less than on the contralateral side (mean 22.6 mm, SD 2.5 mm) with p = .002 by Wilcoxon rank test.

Taylor et al. [42] evaluated prone joint springing for fixation in the thoracic spine and found a moderate agreement (κ = 0.48) with tenderness on skin rolling to determine spinal joint dysfunction within one vertebrae above or below the level of the tender point. In a report by Downey et al. [92], three pairs of examiners attempted to identify the symptomatic segment through posterior-to-anterior pressures. While examiners showed fair agreement (κ= 0.37) in locating segments (± 1 segment), they were less reliable in agreeing on the name of the segment (κ= 0.09), introducing an alternative source of error. Agreement of thoracic springing with provocation of pain was fair to moderate in the work of Cleland et al. [39], depending on spinal level. Similarly, inter-rater reliability ranged from fair to substantial [39,94], with symptomatic subjects producing more reliable responses (κ ≥ 0.6) [95]. Maher and Adams [34] found manual spinal segment stiffness intra-class correlation coefficients higher (0.67 ≤ ICC ≤ 0.72) when pain provocation was considered, but dropped dramatically (0.03 ≤ ICC ≤ 0.37) without pain provocation.

Studies of palpation of the cervical spine are less common. The work by Hanten et al. [49], a lower quality study, used subjects who met the International Headache Society criteria for cervicogenic headache. A panel of 15 cervical mobility and palpation tests was studied for reliability using two examiners. The inter-rater agreement for tests that exceeded κ > 0.50 included: cervical protraction and retraction, both with and without overpressure, and pressure sensitivity paraspinally at C1 and C2. The mean number of positive findings per subject was 3.50, with a Spearman rho =0.943 over two days of testing. However, mixed results were described for overpressure testing (−0.09 ≤ κ ≤ 0.46) by others [97]. Comeaux et al. [93] and McPartland and Goodridge [46] found cervical stiffness testing to have fair to moderate in reliability.

Recommendation: Unclear–high quality evidence suggests moderate validity for the concept of intersegmental restrictions. There is a mix of studies reporting low to substantial reliability for manually locating a site within one segment.

### Stiffness- instrumented

[Quality scores: Validity 0 low, 0 moderate, 0 high; Reliability 2 low, 1 moderate, 0 high].

Few mechanized methods for evaluation of stiffness are accessible in general practice. Three studies [98-100] investigated the usefulness of identifying joint stiffness by instrumentation to locate the site of care. The work of Latimer et al. [100], with a laboratory based instrument, is notable as it observes that the level of stiffness detected is a function of magnitude of the application force. Similarly, the more stable measures of effective stiffness arise from applied forces above 50 N (11.25 lbs).

Leach et al. [98], in a study ranked as being of moderate quality, measured inter- and intra-examiner reliability using a commercially available instrument (Pulstar™) which provides impulse force to the spine above the 50 N threshold for stable measures. Two examiners evaluated 18 healthy 20-to-25 year old subjects for patterns of stiffness along the spine. Good agreement was observed in the pattern of stiffness measures along the length of the spinal column. Inter-examiner ICC=0.87 while intra-examiner findings were 0.78 and 0.89 yielding good to excellent results for reliability.

The work of Owens et al. [99] was rated as low quality evidence. Using a custom built device, posterior-to-anterior spinal stiffness of the lumbar spinal segments was assessed by 9 examiners in low back pain patients. The ICC for inter-rater reliability was very good at 0.79. A quantitative stiffness value of 11.2 N/mm (±3.5) was found, although no differences between segments or comparisons with healthy subjects were available.

Recommendation: Favorable with limitations based on low quality evidence. Limitations are based on instrument availability and uncertainty concerning the generalizability of results to the broader population.

### Static palpation

[Quality scores: Validity 0 low, 0 moderate, 2 high; Reliability 4 low, 7 moderate, 4 high].

The identification and evaluation of relative position of topographical or percutaneous landmarks has long been a part of health care practice. Of the six high quality studies, two evaluated validity [101,102] and four assessed reliability [103-106]. Seven more moderate quality studies [94,107-112] and four low quality studies [113-116] also addressed reliability.

The work of Jende and Peterson [101] utilized what may be termed a ‘proof-by-contradiction’ approach to evaluate seated palpation of C1. Posing an alternative explanation for palpatory prominence as originating from osseous asymmetry (±2 mm), the authors compared the lateral prominence of C1 on palpation in 47 patients against radiographic measures. The ICC for repeated measure of atlas transverse length on x-ray was 0.93. In 57% of cases with laterality on palpation, radiographic measures were equal or more prominent on the opposite side, suggesting that palpatory laterality was not due to an anatomical variant. Unfortunately, no clinical information was reported.

The validity and reliability of locating spinal levels and pelvic landmarks have been reported in six studies [102,108,109,111,112,116] using various means including radiographs to quantify site identification error. Examiners differed in localization of landmarks across a range from 0.5 cm to 2.5 cm, (0.28 ≤ κ ≤ 0.98), often reporting findings from adjacent segments.

Fryer et al. [104] evaluated the intra-examiner and inter-examiner reliability of trained and untrained osteopathic students palpating symmetry in anatomical landmarks (anterior and posterior superior iliac spines (ASIS and PSIS), medial malleoli, sacral inferior lateral angle (SILA) and performing the seated flexion test (SFL). Inter-examiner agreements (kappa scores) for the trained versus untrained examiners were: medial malleoli, 0.31 vs 0.28; ASIS, 0.24 vs −0.01; SFT, 0.14 vs 0.07; PSIS 0.08 vs 0.15; SILA 0.04 vs −0.01. Others [106,115] have followed similar protocols with essentially the same results. Holmgren and Waling [105] and Binkley et al. [107] explored inter-examiner reliability for identifying segmental levels (−0.03 ≤ κ ≤ 0.69, across studies), with comparable results. The prone posture was shown as the more reliable patient positioning for locating spinous processes (inter-examiner agreement 69 < κ < 81) by Byfield and Humphreys [114].

Special cases of static palpatory findings have been studied by others [94,103,110]. In a high quality study, intra-examiner agreement on skin fold thickness and compliance were mixed (−.41 ≤ κ ≤ .23; 0.25 ≤ ICC ≤ 0.28) [96]. Similar results were found by Moran and Gibbons [110] in evaluating ICCs for cranial rhythmic impulses. Conversely, agreement on the identification of the piriformis muscle and iliolumbar ligaments has been reported as substantial (0.61 < κ < 0.87).

Recommendation: Based on high quality evidence, the validity of palpation for localizing the site of care is unclear. A recommendation of favorable with limitations, depending upon the target structure, is made for reliability in localizing common anatomical landmarks.

### Motion palpation

[Quality scores: Validity 1 low, 0 moderate, 8 high; Reliability 8 low, 9 moderate, 11 high].

The underlying premise of motion palpation, particularly with segmental dysfunction of the spine, is that there may be abnormal patterns in relative motion. That the response to manipulation may be related to the degree to which segments are more or less stiff has been shown by Fritz et al. [89]. Motion palpation uses examiner guided motions to manually monitor the relative displacement of bony landmarks through the skin surface. Motions are categorized as being limited/restricted; excessive/unstable or aberrant, suggesting a deviation in path at some point within the range of motion.

Nine studies have assessed validity of motion palpation alone [89,117-121], twenty-five studies have assessed reliability alone [47,50,72,74,104,107,122-139], and three studies have assessed both reliability and validity [140-142].

Humphreys et al. [120] evaluated motion palpation in asymptomatic volunteers with congenital block vertebrae (C2-3 or C5-6) in the cervical spine. Substantial overall agreement (C2-3, κ = 0.65; C5-6, κ = 0.76) was found for identification of the site of greatest hypomobility. Sensitivity ranged from 55% to 78%, greater for the C2-3 level, with specificity that was high (91–98%) for both. Ogince et al. [121] assessed cervicogenic headache patients and asymptomatic controls for C1/2 dysfunction using seated flexion-rotation tests. Blinded examiners identified dysfunction with 91% sensitivity and 90% specificity. Abbott et al. [118] tested the validity of passive physiologic intervertebral motion (PPIVM) of the lumbar spine in recurrent/chronic low back pain patients. Flexion/extension movement of vertebral segments, induced by flexion of the thigh on the pelvis in a side-lying posture, was compared to flexion/extension radiographs. Abnormal motion was defined as a range of normalized movement (segment range/region range) beyond 2 standard deviations (+/−) for a given level in comparison to a sample of healthy subjects. Inter-rater reliability of the radiographic measures, to set the gold standard, ranged between 0.83 ≤ ICC ≤ 0.99). Motion palpation was highly specific (99.5%) for increased translation but was very poor for sensitivity (5%). Likelihood ratios (7.1) for low back pain were significant only for translation in extension.

Landel et al. [119] evaluated a slow application of posterior-to-anterior force to the lumbar spine under MRI imaging, and separately by two examiners, attempting to localize the least and most flexible segments. The study reported substantial and fair inter-examiner reliability of the least (κ = 0.71) and most (κ = 0.29) mobile segments, respectively. Similarly, Abbott et al. [118] looked at posterior-to-anterior translation in response to pressure, with flexion and extension x-rays as the gold standard. Translation was found to be significantly associated with recurrent/chronic low back pain (p < 0.05), with specificity of 89% but poor sensitivity of 29%. Subjects with a positive test had a likelihood ratio of 2.52. The opposite conclusion was found for flexion where specificity was high (99.5%) but sensitivity was low (5%).

Using lateral recumbent patient posture during tests for passive intersegmental motion, the agreement between abnormal flexibilities and pain provocation at the same level was substantial (0.67 < κ < 0.71) for Lundberg and Gerdle [47]. Inter-rater reliability for abnormal motions alone was moderate to substantial (0.59 < κ < 0.75). Phillips and Twomey [142], rated as high quality evidence, compared analysis of lumbar passive segmental movement, coupled with pain provocation response, and results from anesthetic blocks obtained retrospectively in one group and prospectively in another. Sensitivity was 60%, with specificity of 100% in the retrospective group, and 94% sensitivity with 100% specificity in the prospective group. Others have had much less promising results.

Reliability results for motion palpation, absent a gold standard, have been inconsistent. Three studies reported moderate agreement (κ ≥ 0.40) and three reported fair agreement (κ <0.40). Haas et al. [126] evaluated manual end-play palpation of the thoracic spine and found a moderate intra-examiner reliability (κ = 0.5) but only slight inter-examiner reliability (κ = 0.14). Christensen et al. [50], Brismee et al. [132,140] and Smedmark et al. [129] reported fair (0.22 ≤ κ ≤ 0.24) [50] to substantial agreement (κ ≤ 0.65) [132,140]. Others [74,107,117,132,141] have had less impressive outcomes, with mixed agreements on passive segmental flexibility (−0.02 ≤ κ ≤ 0.26, across studies) in the prone position. In asymptomatic subjects [125] agreement between examiners of the cervical spine has been no greater than chance.

Qvistgaard et al. [136] assessed both the intra and inter-examiner reliability of two experts using the stork test, pelvic girdle rotation, and the lumbar spring test to identify dysfunctional lumbar spinal levels. The study considered two definitions of reliability; perfect match (PER) defined as positive results on the same segmental level, and acceptable match (ACC) defined as positive results within one segmental level. The study found moderate and substantial levels of intra-examiner reliability (PER, κ = 0.60; ACC, κ = 0.70) with fair and moderate levels of inter-examiner reliability (PER, κ = 0.21; ACC, κ = 0.57). The work of Love [123] and Mootz et al. [124] indicated only chance agreement.

Robinson et al. [137] found near perfect inter-examiner reliability among expert examiners assessing passive sacroiliac joint play (0.78 ≤ κ ≤ 0.88). Similar results were obtained by Hungerford et al. [135] (0.67 ≤ κ ≤ 0.77). In contrast, Schneider et al. [138] reported mixed results (− 0.17 ≤ κ ≤ 0.17) for two experts utilizing a segmental mobility test to assess normal or restricted mobility across the lumbar spine and sacroiliac joints. Fryer et al. [104] looked at the seated flexion test for sacroiliac fixation finding slight levels of inter-examiner reliability (κ = 0.14). Vincent-Smith and Gibbons [128] had better results with the standing flexion test (κ = 0.052). Arab et al. [72] constructed composite tests from motion palpation (standing flexion, seated flexion, Gillet, prone knee flexion test, leg length) and provocation manoeuvres (Patrick-Fabre, thigh thrust, resisted abduction). With positive findings on three or more of the palpation tests and two or more of the provocation manoeuvres, the inter-examiner agreement was substantial to excellent (prevalence-adjusted and bias-adjusted kappa; 0.52 ≤ κ ≤ 0.92). Meijne et al. [127], Tong et al. [133] and Carmichael [122] found the same manoeuvres individually less reliable (−0.32 ≤ κ ≤ 0.27, across studies).

A few investigators have attempted to identify procedural characteristics that improve inter-rater reliability. Marcotte et al. [130] suggest that the orientation of the examination input force, but not amplitude of force [131] may increase reliability (from κ =0.34 to κ = 0.68). Cooperstein et al. [139] found that high examiner confidence raises agreement to κ = 0.82.

Recommendation: Favorable with limitations (region of the spine, direction of movement and method employed), based on high quality evidence for both validity and reliability for use in localizing the site of care.

### Leg length inequality (LLI)

[Quality scores: Validity 1 low, 1 moderate, 4 high; Reliability 6 low, 4 moderate, 6 high].

Much attention has been given to the question of relative leg length and change in apparent relative leg length as an indicator for site of treatment. Six articles pertaining to the validity of measures of LLI [143-148] and fourteen articles pertaining to the reliability of LLI assessment [106,108,148-159] were retrieved and reviewed. Most of the studies assessed LLI with the subject in the prone position, but some had subjects in the standing or supine position.

The construct validity for an etiological role of LLI in lower quadrant and low back dysfunction is controversial. Motion, alignment and muscular endurance of the low back and pelvis are altered in the presence of leg length differences [160], yet cross-sectional studies [160-162] fail to show a relationship between LBP and LLI alone. At the same time, in a 4 year prospective study of 136 students, Twellaar et al. [161] found pelvic obliquity to be associated with injury rates including backache. Knutson [146] measured leg length inequality among selected volunteers who were partitioned according to their history of recurring low back pain. Pain scales were higher (p < 0.001) in subjects with length asymmetry, with sensitivity of 87% and specificity of 84%.

Petrone et al. [148] found similar validity and reliability of standing evaluations using the PALM™ assessment of iliac crest level using a gold standard of standing pelvic radiographs with the central ray at the level of the femoral heads. The ICC for inter-rater reliability was 0.97. The agreement between tape measure assessment (ASIS to the medial malleoli and scanogram) fared less well with 0.359 ≤ ICC ≤ 0.770.

Hinson et al. [152], Woodfield et al. [159], Kmita and Lucas [106], and Fryer [156] found supine visual leg checks to show varying degrees of inter-examiner reliability. Cooperstein et al. [163] evaluated the validity and reliability of prone compressive leg length assessment using standardized foot wear containing shims of various thicknesses in one shoe. Artificial leg length differences over 3.7 mm were reliably identified with an intra-examiner ICC of 0.85. Fuhr and Osterbauer [149], Nguyen et al. [153], Schneider et al. [157], and Holt et al. [158] used prone compressive leg length estimates of volunteers, finding substantial reliability (0.65 ≤ κ ≤. 0.70).

Some clinicians follow prone compressive leg length checks with knee flexion to 90 degrees in order to reveal change in relative length. Schneider et al. [157] found this maneuver to result in a high prevalence (95%) of increased length on the short leg side, confounding calculation of the kappa statistic. Holt et al. [158] were able to define reliability with knee flexion at ĸ = 0.65. Low level evidence from Eriksen [145] examined change in leg length with specific challenge to a vertebral level on an unspecified number of subjects. Of 18 variables, only one had a notable concordance (κ < 0.40) between the change in LLI and x-ray findings.

Blocking methods insert varying sized blocks/lifts under the feet to level the standing pelvis or to simulate LLI. The method investigated by Gross et al. [151] was less accurate (±0.46 cm) and of lower inter-rater reliability (ICC=0.77) than prone compression or the PALM™ method. Hanada et al. 2001 [154] found nearly identical results, comparing block height with scanogram measures. Gibbons et al. [155] assessed the ability of examiners to palpate the iliac crest levels on subjects who had an artificially created asymmetry in standing leg length, finding that they were unable to reliably detect discrepancies of l cm or less.

Three reports were identified which met the inclusion criteria and specifically addressed the question of leg length as an indicator for the site of care. Montgomery et al. [144], in a low quality validity study, reported substantial agreement (κ = 0.664) between palpatory findings of unilateral sacral prominence suggestive of pelvic torsion and radiographic findings of leg length difference. Using variants in positioning of the extremities and head, leg length has been offered as a means to identify the site for necessary care. Younqvist et al. [150] evaluated this maneuver for the C1 segment, reporting a κ = 0.52. In contrast, Schneider et al. [157] evaluated head positioning in subjects with leg length difference ≤ 0.25 inch with mixed, but poor reliability (−0.02 ≤ κ ≤ 0.04).

Recommendation: Favorable with limitations for assessing the pelvis, based on high quality studies. Validity for relationship to symptoms has not been demonstrated. Reliability appears method-dependent.

### Manual muscle testing

[Quality scores: Validity 1 low, 0 moderate, 1 high; Reliability 3 low, 3 moderate, 1 high].

Manual examination of the capacity to produce powerful contraction by muscle is a classical part of the examination for neuromotor control. Traditionally, it has been used as a graded system [164] of testing individual or isolated groups of muscles related to a specific function (e.g. deltoid for abduction). In this role, muscle testing may provide information consistent with the myotomal connections, potentially indicating nerve root involvement. Its use has been extended from assessing frank neuromuscular pathology to determination altered function which is not necessarily pathological [165-167].

Nine papers were identified within the inclusion criteria [33,39,41,165,167-171].

Used to grade strength, McCombe et al. [41] found inter-rater reliability for motor power in the lower extremities was substantial to perfect (0.65 ≤ ĸ ≤ 1.0) for one pair of examiners, but only poor to moderate (0.02 ≤ κ ≤ 0.35) for another pair. The study by Viikari-Juntura et al. [33] in neck pain patients, found agreement on muscle atrophy was fair to substantial (0.35 ≤ κ ≤ 0.81) while reliability for grading muscle strength was moderate to substantial (0.40 ≤ κ ≤ 0.64). Cleland et al. [39], in a limited sample of muscle tests, found inter-rater agreement to vary depending on the muscle being tested, with overall agreement ranging from 41% to 91%.

Used as an indicator of altered function, not necessarily pathological, the validity of muscle testing was evaluated by Ludtke et al. [170] in a double blind study with 4 examiners using 7 volunteers with confirmed allergy (IgE production) to wasp venom. The venom or a saline solution vial was randomly placed below the umbilicus, and change in muscle strength (anterior deltoid) was evaluated. The correct assessments of placebo versus allergen were 60% and 40%, respectively. Inter-rater reliability overall was κ = −0.01, suggesting less than chance agreement. Calderon and Lawson [165] found a highly mixed inter-rater reliability, based on which muscle was being isolated for testing (−0.07 < κ < 0.90). The same data was replicated (for purposes of this report, scored only once) in a publication under Lawson and Calderon [166]. Patient initiated strength by dynamometry [168] has been shown to have substantial reliability (κ = 0.96–0.99) whereas doctor initiated testing showed lower reliability (κ = 0.55–0.76). Haas et al. [169] evaluated the reliability of muscle testing in response to a provocative challenge attempting to localize the vertebral site needing treatment in both symptomatic and asymptomatic subjects. A 5 kg force directed to produce rotational stress was applied to thoracic spinous processes, preceded and followed by a manual muscle test of the strength in the piriformis muscle. Inter-rater reliability approached zero (κ = −0.04, symptomatic; κ = −0.02, asymptomatic) indicating agreement by chance alone. Caruso and Leisman [171], in a low quality study, suggest that accuracy in functional muscle testing improves with years of clinical experience. While reliability was claimed, no kappa or ICC values were presented.

Pollard et al. [167] took a novel approach asking whether a change in muscle strength (deltoid) following identification of palpatory tenderness over the lower right quadrant (ileocecal valve/McBurney’s point) may be related to the presence of low back pain. While one cell of the 2x2 table was underpopulated, the authors calculated a sensitivity of 0.86 and specificity of 0.97.

Recommendation: Favorable, with a moderate level of evidence, for strength grading to localize nerve/nerve root levels. Unfavorable as a diagnostic indicator of non-pathological altered function leading to localizing site of care.

### Range of motion

[Quality scores: Validity 1 low, 0 moderate, 3 high; Reliability 12 low, 8 moderate, 2 high].

Many studies have investigated range of motion in healthy and unhealthy subjects. Five high quality studies [54,172-175], eight moderate quality studies [33,39,97,111,176-179] and twelve low quality studies [41,180-190] which met the inclusion criteria for this review were identified. Four assessed validity [48,172,175,182] and the others assessed reliability.

The construct validity of reduced range of motion in painful disorders has been examined by several groups. Viitanen et al. [172] correlated different spinal ranges of motion (ROM) and the results of 17 repeated tests with spinal radiological changes in 52 male patients with ankylosing spondylitis. Inter-rater reliability on motion measures was good to excellent (0.84 ≤ ICC ≤ 0.98). Osterbauer et al. [182] measured kinematic parameters of head motion during tracking tasks (flexion, extension, etc.), and cervical ROM was measured via a head mounted optoelectronic inclinometer. Pain and disability were assessed via the neck disability index questionnaire and visual analog pain scale. A scoring system of kinematic abnormalities was created ranging from 0 to 3. A cutoff of ≥ 0.5 correctly identified the greatest number of subjects and minimized false positives (sensitivity 77%, specificity 82%, likelihood ratio 4.5). ROM performed similarly well at a cutoff of 1 SD below the normative mean (sensitivity 77%, specificity 84%, likelihood ratio 3.9). Hall and Robinson [177] also found a strong correlation (r = 0.8) between severity of headache and restriction, with excellent reliability (0.92 ≤ ICC ≤ 0.99) in measuring neck motion. Mayer et al. [182] examined reliability and responsiveness of inclinometric measures of rigidity at intervertebral levels during lumbar flexion/extension and lateral bending in seventy chronic low back pain patients. Four had measurable rigidity at one level, thirty-six at two, and thirty at three levels. Repeatability was assessed, with high correlation in flexion/extension (0.90 < r < 0.97, p < 0.01) and moderate correlation for lateral bending (0.65 < r < 0.95, p < 0.05). Patients were assigned to one of two treatment groups: exercise only, or exercise with localized joint anesthesia. Pain intensity (VAS) and ROM improved with treatment and patients who received joint anesthesia showed greater improvements in ROM. Leboeuf-Yde and colleagues [48] evaluated the range of motion in 166 individuals, 46 of whom had never had low back pain, 18 of whom had pain on the day of examination, the remainder having had a history of pain sometime within the past year. Sensitivity for “low back pain today” was low, ranging from 22% to 44% in a hierarchy of rotation < flexion < side bending < extension. Specificity fared better (range 98% to 100%) with side bending < extension = flexion = rotation. All tests reported high levels of positive predictive value (range= 86%-100%) and negative predictive value (range= 77%-90%). A novel description of the quality of movement, “gearbox flexion” denoting an uneven path of motion, was evaluated. This type of movement was highly specific (1.0), with strong positive (1.0) and negative (0.73) predictive values, although the finding had very low prevalence.

Goniometric devices of several kinds have been studied [111,175,180,182-186,188,190]. These devices include gravity and electronic goniometers, optoelectronic monitoring of body markers and tape measure approaches. In general, the reliability of measures has been good to excellent (0.72 ≤ ICC ≤ 0.995). Several special circumstances have been noted with range of motion measures. Haynes et al. [186] observed degradation in repeated measures with forward head tilt of up to 10 degrees. Lateral neck flexion was only fair in agreement for Hoppenbrouwers et al. [178]. Using a tape measure [41,172,190] to monitor landmark displacements is comparable to goniometry in reliability. Whether active or passive neck ROM is measured, extension may be more reliable (ĸ ≥ 0.85) than flexion (κ ≥ 0.33). Piva et al. 2006 [85] found substantial and almost perfect levels of inter-examiner reliability (0.78 ≤ κ ≤ 0.94) in examining the neck, while Viikari-Juntura et al. [33] reported lower levels of reliability (0.40 ≤ ĸ ≤ 0.56). Also for the neck, Cleland et al. [39] showed good reliability (0.57 < ICC < 0.78) for measuring range of motion, and moderate to substantial kappa agreement in eliciting self-report of symptom aggravation with specific motions. Maigne et al. [54] found moderate agreement (κ = 0.57) when examiners classified patients as being slightly, moderately or severely restricted.

Recommendation: Favourable for use to localize the site of treatment within a spinal region, based on high quality evidence for validity and reliability.

### Tissue temperature, texture, and tone

[Quality scores: Validity 0 low, 0 moderate, 5 high; Reliability 5 low, 4 moderate, 4 high].

Modern thermography uses infrared sensing devices to evaluate the relative levels of heat emitted through the skin, while thermometry uses direct skin contact via thermocouples. The temperature of tissues is determined by a number of factors, including metabolic activity, perfusion and environmental temperature. Two papers [191,192] suggest that while thermometry may provide valid measures of skin temperature, stringent environmental control is necessary to achieve stable readings. Readings are perturbed by the skin contact accompanying spinal manipulation, at least with instrumented methods, and the responses to manipulation are multiphasic, confounding interpretation.

Four additional studies met the inclusion criteria for examining the validity of skin temperature [193-196] to identify the site for application of treatment. Chafetz et al. [193] evaluated a small sample of patients with confirmed L4/5, L5/S1 root distortion on CT scans versus healthy controls. The authors reported specificity of 60% and sensitivity of 100% in thermographic results. McCulloch et al. [194], in somewhat larger groups, reported sensitivities for two examiners at 60% and 50%, and specificities at 45% and 48%, using patients with clinical sciatica, and CT or MRI results as the gold standard. More recently, Zaproudina et al. [196] evaluated the side-to-side differences in temperature of the plantar surface in chronic low back pain patients. Differences were observed between patients with low back pain only versus those with referred leg pain (Mann–Whitney test p < 0.05). The severity of Oswestry scores for current LBP disability level correlated with the magnitude of temperature differences with coefficients of correlation of 0.502 (p = .000). There were also correlations between magnitude of temperature asymmetry and both straight leg raise (p < 0.005) and side bending motion (p < 0.05). A study which was ranked as providing low quality evidence [195] reported poor to moderate correlation between thermography and palpatory findings of segmental restrictions, defined by tenderness, positive skin rolling and motion palpation.

Plaugher et al. [197] which was ranked as providing high quality evidence, reported inter-examiner reliability ranging from slight to substantial (0.03≤ κ ≤ 0.65) and fair to substantial intra-examiner reliability (0.03 ≤ κ ≤ 0.66,) depending upon the region of spine. Two other studies, ranked respectively as of moderate [40] and low quality [55] found slight to moderate agreement (0.0 < κ < 0.63) of paraspinal skin temperature measures. Hart et al. [198] attempted reliability testing with 10 minute intervals between samples with good ICCs of > 0.75, while Owens et al. [199] reported excellent ICCs using a handheld thermographic device (0.918 < ICC < 0.975). The inter-rater reliability of mastoid fossa temperature measurement was also good (0.671 < ICC < 0.748) [200]. Finally, Owens et al. [199] were able to obtain excellent reliability when repeated measures were obtained over 3 minute intervals.

Five papers evaluated tissue texture assessment. Differences in response to skin rolling have been claimed to be a function of surface/subsurface texture (e.g. Diakow et al. [195]). Tests using skin rolling, however, do not have any independent measure of texture. In a variation of classical palpation, a high quality paper compared pain production on skin rolling with pressure algometry readings. A highly significant (p < 0.0005) decrease in pain threshold tolerance at the level of tenderness on skin rolling was found in comparison to non-tender control points in the thoracic spine [42]. Degenhardt et al. [201] defined a positive test for tissue texture as the presence of localized edema or fibrotic changes. After an interval of consensus training for 2 examiners, agreement on tissue texture was moderate (ĸ=0.45). McPartland and Goodridge [46] used palpatory sense of ‘fullness’ over articulations of the upper cervical spine, rated on a 10 point scale, with only slight agreement (ĸ =0.19) but had a low prevalence which may have artificially reduced the kappa score. A more clear definition of texture was given by Paulet and Fryer [202] as an abnormal hardness, bogginess, or ropiness of the underlying paraspinal muscles. Inter-examiner agreement for the site with the most marked tissue texture changes was fair (k = 0.26).

Recommendation: The evidence from studies with high validity and reliability is favorable for the use of thermography/thermometry of the lower limb in confirming frank sciatica. The evidence from high quality studies is unfavourable toward the use of paraspinal skin temperature measures to locate the site of care, due to limited reliability. Evidence of moderate quality is favourable toward the use of skin rolling and palpatory assessment of tissue texture, although the relationship of skin rolling to tissue texture is uncertain.

### Specialized tests

#### Current perception threshold

[Quality scores: Validity 0 low, 1 moderate, 2 high; Reliability 0 low, 2 moderate, 0 high].

Current perception threshold (CPT) is a measure of peripheral sensory nerve sensitivity to surface electrical stimulation and was introduced by Rendell et al. [203] in the management of diabetic sensory neuropathy. Segmental specificity of CPT for lumbar radiculopathy was validated by Yamashita et al. [204] and Imoto et al. [205]. Tests demonstrated significant differences (p<0.01) in sensory function between healthy volunteers and patients with unilateral disc herniation confirmed by magnetic resonance imaging and pain within the distribution of the corresponding lumbar nerve root (L5 or S1). Attesting to the validity of CPT in identifying the level of neuropathy, Imoto et al. [205] demonstrated that following microdiscectomy, 66% of cases showed significant improvement in CPT (p<0.05), along with symptomatic improvement. Patients who did not show improvement in symptomatology, as a cohort, did not show any significant change in CPT.

Recommendation: High quality evidence is favourable for the validity of using CPT to identify the segmental level of frank neuropathy. No evidence which met our inclusion criteria addresses the validity or reliability of CPT in otherwise localizing the site for manual treatment.

### Galvanic skin response

[Quality scores: Validity 0 low, 1 moderate, 0 high; Reliability 1 low, 0 moderate, 0 high]

Galvanic skin response (GSR) is the measure of electrical conductance of the skin and in the short term is most strongly influenced by the rate of sweat secretion, since higher moisture content improves electrical conductance. Because sweat secretion is modulated by the sympathetic nervous system, GSR has sometimes been used as an indirect measure of changes in sympathetic nervous system activity, as for example occurs in some instances of psychological or physiological stress. Two articles concerning GSR satisfied the inclusion criteria [206,207].

Nansel and Jansen [206] were unable to validate GSR changes by examining concordance with findings on manual palpation. While they did not publish their kappa values, reliability was low. Plaugher et al. [207] reported moderate to substantial interexaminer reliability in a study which was rated as low quality.

Recommendation: The evidence from a small number of studies of low to moderate quality is unfavorable, in terms of both validity and reliability, for the use of GSR in determining the site of care.

### Surface electromyography (SEMG]

[Quality scores: Validity 0 low, 1 moderate, 3 high; Reliability 2 low, 1 moderate, 1 high].

Three recent reviews of the literature have evaluated the validity [208,209] and reliability Danneels et al. [210] of SEMG as a tool for identifying subjects with back pain. SEMG results vary widely [208,209] based on test methodology. Multiple technical factors complicate the interpretation of results including the choice of electrodes, the variation in skin impedance from site-to-site, the site of electrodes in relation to muscle motor points, the signal normalization method, the temperature of the muscle and skin, the fat layer thickness and the presence of postural support. While SEMG does not reliably isolate the activity of a specific muscle [208], the results of intramuscular EMG and SEMG seem correlated with each other [209].

Geisser et al. [208] conducted a meta-analysis of studies evaluating SEMG in static posture, isometric tasks, and myoelectric response to expected or unexpected sudden loads. Five studies showed no difference between healthy and unhealthy subjects in upright posture, including no difference in side-to-side asymmetry, except during forward flexion. Another five showed higher SEMG amplitudes for static upright postures in low back pain patients than in controls. Patients with disc disorders had higher amplitudes during seated SEMG tasks than normals and other LBP patients. No group differences were observed when SEMG was measured while subjects were lying prone or sitting unsupported. Studies consistently found a lower flexion-relaxation response among subjects with LBP with a large effect size, and good sensitivity (88.8%), specificity (81.3%) and responsiveness to treatment.

Within the context of the present study, only six additional studies met the inclusion criteria for examining the validity and reliability of using SEMG to target sites for spinal manipulation. Dankaerts et al. [211], found differences between healthy subjects and subgroups of chronic low back pain patients (p<0.001). However, the myoelectric differences were dependent upon clinical pre-classification into groups according to postural habits. Fryer et al. [212] demonstrated resting paraspinal muscle variation in the thoracic spine with poor to moderate repeatability using either intramuscular (0.55 ≤ ICC ≤ 0.94) or surface (0.35≤ ICC ≤ 0.69 ) electrodes. Under conditions of maximal voluntary contraction, however, both methods of measure had increased reliability (κ ≥ 0.91). When EMG measures were partitioned between sites considered by two experienced clinicians as being normal to palpation versus sites with local abnormality in tissue texture and tenderness, no statistical differences were found for either intramuscular (F1,14=3.18, P=.10) or for SEMG electrodes (F1,18=1.53, P=.76). A study ranked as high quality [213], examined the reliability and validity of surface EMG in identifying the laterality and segmental level of dysfunction in patients with low back pain. Repeatability of measures was acceptable over several days in pain free subjects (ICC > 0.75). However, static tests showed no ability to identify the laterality or segmental levels of tenderness to palpation in subjects with back pain. A study rated of low quality found that inter-examiner agreement was mixed (0.20 < ICC < 0.55; -0.13 ≤ κ ≤ 0.59) and unacceptable for clinical application [55].

Leach et al. [214], in a study consistent with the review by Geissler et al. [208], examined a small sample of acute LBP patients and control subjects without neurological deficit. Robust group differences were seen in flexion-relaxation between groups, negatively correlated (−0.74 < r < −0.50) with straight leg raising, and were responsive to treatment, improving in terms of disability as measured by the Oswestry scale (r = .42).

Recommendation: The current evidence, which is of high quality, is favorable with limitations towards the validity and reliability of using SEMG to identify cohorts of patients with abnormal neuromuscular control, such as an altered flexion-relaxation response. However, evidence of moderate quality is unfavourable toward the use of SEMG in localizing treatment to a specific site.

### Radiographic imaging

[Quality scores: Validity 1 low, 1 moderate, 5 high; Reliability 5 low, 5 moderate, 1 high].

The validity of computer modeling for differentiating normal from symptomatic cases based on x-ray digitization of the lumbar and cervical lordoses has been studied by the Harrison group [215,216]. X-ray digitization itself has shown inter-examiner reliability for three examiners ranging from 0.71 to 0.99, depending on the measure [217,218]. Differences in ellipse parameters fit to spinal curvature and some standard radiographic measures demonstrated significant (lumbar, 0.0001 ≤ p ≤ 0.0113; cervical, 0.0001 ≤ p ≤ 0.05) differences between groups of healthy and unhealthy subjects. Careful repeated positioning of patients for x-ray can be achieved [219] with no significant difference in measures. Radiographic measures of sacral angle show poor correlation with measures taken by an inclinometer placed externally over the sacrum [220]. However, none of these measures have been shown to have predictive value for assessing individual patients.

Abbott et al. [118], examined the hypothesis that palpable intersegmental hypermobility correlated with hypermobility revealed in lateral flexion-extension radiographic studies in chronic low back pain patients. The authors concluded that, overall, the clinical examination procedures displayed moderate validity in comparison to radiography, with palpation of translation in the sagittal plane faring better than rotation. Continuing on this theme, Abbott and colleagues [221] looked at the prevalence of radiographic movement disorders related to low back symptom status. Using flexion-extension lateral radiographs from cohorts of healthy and chronic/recurrent low back patients, the relative contribution of each segment’s motion to the regional motion was computed. Lumbar segmental movement disorder was defined by 2 methods: i) motion beyond 2 standard deviations from the mean of a healthy, control cohort and ii) a normalized, within subject measure of segmental motion beyond 2 standard deviations from the mean of the relative contributions of each lumbar segment. The latter measure was more sensitive, but both identified significantly more instances of lumbar segmental movement disorder in the low back pain patients than in the health controls. Lumbar spine translational rigidity (as opposed to rigidity in rotation or instability in general) was associated with higher disability scores (p=0.010) Again, the predictive value for individual patients has not been determined.

Independent radiologists [118] have evaluated the concurrent validity and reliability of manual tracings and computer generated measures of sagittal rotation (ICC = 0.98) and translation (ICC=0.98). Harrison et al. [222] repeated x-rays of subjects at intervals of 3 to 12 months. Follow-up measures in control subjects were similar in the mean to baseline measures; however, no reliability statistics were performed. Jende and Peterson [101], in a high quality validity study with a small nested intra-examiner reliability study took bone geometry measures from the same radiographs on separate occasions, giving an ICC = 0.93. Similarly, two examiners digitized x-ray landmarks twice [215], obtaining ICCs for intra-examiner and inter-examiner that were described as “high” with standard error of measure < 2%.

Reliability of radiographic markings from the Gonstead system of analysis was tested by Plaugher and Hendricks [223]. Examiners were independent and, for the study of intra-examiner reliability, blinded on their second evaluation with respect to findings on their first evaluation. Intra-examiner scores (0.846<ICC<0.999) tended to be minimally higher than inter-examiner scores (0.812<ICC<0.995). Antos et al. [224], in a paper ranked as of low quality found strong reliability (κ =0.80) for assessing flexion of C4/5, by videofluoroscopy, as the increase in spinolaminar distance between segments. Higher concordance was found for radiologists more experienced in fluoroscopic interpretation [225].

The extensive work of Jackson et al. [84,226] attempted to evaluate the reliability of various measures of alignment in lateral x-rays of the spine, including anterior head translation, atlas plane to horizontal, Ruth Jackson's cervical stress lines, and five relative intersegmental rotation angles for the neck. Inter-rater reliabilities were substantial to near perfect in the cervical spine (0.74 ≤ κ ≤ 0.99). Phillips et al. [227] evaluated 56 radiographic variables demonstrating a high inter-observer reliability for interpretations by chiropractors, but not for medical physicians. Most notably, findings related to disc space narrowing were the few statistically associated with back or leg complaints (P = 0.025).

Lumbar patterns of vertebral displacement on lateral bending films, following the definitions attributed to Grice [228], measuring lateral tilt combined with rotation have been studied for reliability. In this low quality study, inter-rater concordance for lateral tilt was moderate (0.49 < κ < 0.65) for L1 to L4 but fair (0.23 < κ < 0.24) for L5. Reliability for rotation was varied over a broader range (0.19 < κ < 0.60) for all spinal levels depending on pairing of examiners. The ability to predict aberrant lateral bending of a vertebral segment from x-rays taken in the neutral orientation of the segment was studied by Haas and Petersen [229]. Healthy and low back pain subjects were examined. In this high quality study, a moderate negative correlation (−0.39 < r < −0.16) was found between the amount of neutral lateral tilt of the vertebrae and its tendency to increase that tilt when lateral bending to the opposite side. Both symptomatic and asymptomatic subjects revealed the same behaviour.

Olson et al. [230] hypothesized a difference in motion in lateral bending of the upper cervical spine based on different standardized starting postures: neutral or minimally flexed. However, in this low quality reliability study, no difference was observed (p > 0.05).

Rhudy et al. [231] examined agreement on the decision of lesion site based on systems of analysis. Two of those systems, full spine radiography listings and Gonstead, use full spine x-rays, although the Gonstead approach adds information from skin temperature readings. No details of the system analyses were included and comparison of systems in this fashion has little meaning.

Recommendation: Evidence of high quality supports the use of static and motion studies to identify hypermobile segments but not hypomobile segments. Thus, in terms of validity, the high quality evidence available at this time is unfavourable for use of x-rays to determine the site of care.

### Integrated P.A.R.T.S. Studies

[Quality scores: Validity 0 low, 0 moderate, 1 high; Reliability 3 low, 7 moderate ,1 high].

Several reports have utilized a montage or large panel of examination techniques in order to localize the target of care to a region or within a narrow anatomical limit, such as an individual articulation. Ten of these studies [48,68,136,201,232-234] attempted to combine results into a form of summary score to guide the decision to provide care. Two studies [33,71] evaluated neurological tests designed to localize pathology according to sclerotome or dermatome, and orthopedic tests localizing to specific regions/articulations. Results from separate tests were not analyzed statistically to determine their individual contributions to a global decision to treat. Where in fact integrated PARTS studies did analyze the individual contributions of specific tests, these results have been discussed within the appropriate preceding sections.

Leboeuf-Yde et al. [48], in a high quality study, asked examiners to categorize subjects into groups of “low back pain today” and “low back pain never” after using a panel of tests for posture, motion and pain provocation. The accuracy rates were 78% and 67%, respectively.

An early study by Rhudy et al. [231] focused on the tools used to identify the site of care. The inter-examiner agreement findings of three examiners who analyzed full spine x-ray films were compared with their motion palpation findings and each of these were also compared with their Gonstead style analysis of neurocalometer readings on a small sample of fourteen symptomatic patients. The investigators reported almost perfect/substantial correlation only 19% of time, fair/moderate correlation 21% of time and poor/nil correlation 60% of the time. Hawk et al. [232], bundled assessment of segmental hypomobility/hypermobility, tissue texture, palpable temperature, and tenderness elicited on palpation to identify the site of treatment. Agreement was slight, at best.

An inter-examiner reliability study by Keating et al. [235] attempted to correlate eight different tools common in chiropractic practice: i) palpatory pain over osseous structures and ii) paraspinal soft tissues, iii) temperature differences between adjacent segments as measured by the dermothermograph, iv) visual inspection for segmental abnormality, v) active and vi) passive motion palpation, vii) muscle tension palpation, and viii) misalignment palpation. Scores were combined to form a composite joint abnormality index, with osseous pain and soft tissue pain, temperature and visual observation giving the strongest correlations (0.34 < r < 0.65). In a follow-up study that was also rated as moderate quality, Boline et al. [55] assessed the composite from visual inspection, palpation for pain over osseous and soft tissue structures, surface EMG and the dermothermograph. Reliability of the combined scores was mixed (−0.3 < κ < 0.56) and judged unacceptable for clinical application. They again reported that palpation for pain over osseous and soft tissue structures and visual observation of posture produced good to excellent inter-examiner agreement. Results from visual postural analysis, pain description by the patient, plain static erect x-ray films, leg length discrepancy, neurological tests, motion palpation, static palpation and orthopedic tests were evaluated by French et al. [68]. Overall, the measures were not reproducible and the decision to treat, either by different examiners on the same occasion or the same examiner on different occasions, was not reproducible.

Petersen et al. [71] linked a systematic partitioning of patients according to suspected pathoanatomical site of pain production based on pain distribution, response to postural movements and orthopedic maneuver. Inter-rater kappa scores ranged from 0.44 ≤ κ ≤ 1.00 for assignment of patients in groups for discogenic, radicular, zygapophyseal, sacroiliac and myofascial categories.

Qvistgaard et al. [136] focused on identifying abnormal spinal segments (± 1 segment) as the most pronounced dysfunction in the lumbar spine. Testing combined standing (trunk side bending, flexion, ‘stork test’), supine pelvic girdle clockwise/counter clockwise rotation, prone lumbar springing, and side-lying multifidus tension manoeuvres. Kappa values were: segmental diagnosis, 0.57; multifidus test, 0.48; side flexion, 0.45; and ventral flexion 0.44. A similar approach to the thoracic spine was used by Potter et al. [233]. Combining inspection of posture, voluntary movement, passive movement and static palpation, identification of a dysfunctional joint in the thoracic spine showed moderate to poor reliability (ICC = 0.70, C.I. 0.27–0.90). For the lumbar spine, the results were better: ICC = 0.96 (95% CI, 0.87 to 0.99). Toussaint et al. [236] examined the sacroiliac joint with a combination of the standing flexion test, spine test (Gillet), and iliac springing test. Agreement on side, based on a positive result combined for any two tests, was moderate to substantial (0.483 ≤ κ ≤ 0.677).

Degenhardt et al. [201,234] evaluated a consensus training method to enhance the inter-rater reliability of a panel of tests. After training intervention [234] for 12 groups of 3 examiners, agreement on tenderness rose from κ =0.32 to κ =0.68 and that for tissue texture improved from κ =0.12 to κ =0.45. In the separate study [201], training was able to produce increased agreement in static palpation asymmetry (κ =0.18 to 0.59), tissue texture (κ = −0.01 to 0.45), anterior springing (κ =0.29 to 0.44) and palpatory tenderness (κ = 0.32 to 0.65). Retention of the improved skill has not been reported.

Recommendation: Unclear, based on moderate quality evidence, for examination montages contributing any more than their component elements to the decision to localize treatment.

## Discussion

The work of this report represents the most comprehensive review of the literature, to date, in relation to the diagnostic methods used for locating the site of care at which to apply manipulation treatment methods. Despite a number of studies addressing the questions of validity and reliability over the years, the research community’s sense of what constitutes a good study of diagnostic accuracy appears to be evolving. Guidelines for evaluating the strength of evidence are relatively recent in comparison to guidelines for studies of treatment. The first broadly accepted sets of standards for the design of studies of diagnostic accuracy, Standards for Reporting of Diagnostic Accuracy (STARD) and the Quality Assessment of Diagnostic Accuracy Studies (QUADAS), were published in 2003 [25,26,237,238]. The Quality Appraisal tool for Studies of Diagnostic Reliability (QAREL) only appeared in 2010 [27].

Applying these tools to the literature found high quality evidence for the majority of the P.A.R.T.S. constructs. Constrained by the current understandings of manipulation treatment methods and the patients to whom they are applied, better practices, if not best, for practitioners are evident and summarized in Table 3. Very few methods can be used in isolation. Taken as a whole, the literature continues to support the fundamental principles of clinical differential diagnosis [10]. The physical examination should be contextualized by the patient’s history and presenting complaint to progressively narrow the focus of attention, first to region then local site and, sometimes, tissue. The body of evidence seems to support more direct, mechanical methods of assessing and identifying the site of care, and in general is not supportive of less direct methods such as manual muscle testing for nonpathological states, thermography, surface electromyography and measures of electrodermal activity. Fixed examination montages are no more helpful than information derived from the individual components. Manoeuvres that replicate the patient’s familiar pain may be the most consistent sources for diagnostic information. A number of assessment methods were judged to be useful for patient screening or for narrowing the topographical focus of examination. These included postural assessment, orthopedic testing in general, and range of motion testing, as well as assessment of leg length inequality. While there is favourable evidence for a number of palpation methods, there are significant limitations. The inability to locate anatomical landmarks likely is a common underlying feature. When the error in accuracy is taken into account by enfolding those errors (e.g. ± one vertebral segment) into the decision criteria, inter-rater reliability increases substantially.

Examining this error construct in light of more recent work may be instructive. Is the error a result of limitations in communicating what is being perceived by the examiner? May it be a result of the variation in clinical presentations in patients that are believed to respond to manipulation treatments? Future diagnostic studies are likely to benefit from studies on mechanisms of the examination and of the underlying clinical state. For example, the work of Degenhardt et al. [201,234] strives to understand the elements of examiner perceptions and to train examiners to reliably locate them and convey their interpretations. Fritz and colleagues [74,89,90,221] are attempting to tease apart the objective static and dynamic features of spinal function in relation to examination findings and responses to treatment.

The ambiguity of the clinical state of the underlying lesion or lesions treated with manipulation is a constraint on the literature and this constraint cannot be effectively controlled by research methodologies. At present, there is no ideal gold standard of comparison. In response, a pragmatic approach has been taken in this, consistent with the practitioner’s experience, and accepts forms of concurrent, construct and face validity for the necessity of care as derived from the patient’s clinical presentation.

### Limitations

•Although every effort was made to perform an exhaustive and complete search, the abundance of relevant literature, coupled with the fact that some authors did not choose useful indexing terms, guarantees that some literature was missed.

•A number of studies used examiners of doubtful ability–commonly students–and this seems incongruous when investigating what may be complex psychomotor skills, as suggested by the work of Degenhardt et al. [201,234].

•Rules that are used to rate the strength of evidence are by definition arbitrary and thus subject to discussion.

•A number of authors utilized suboptimal methods of data analysis (e.g. correlation analysis) particularly for addressing inter-rater reliability. Found usually in the lower ranked articles, correlation analysis may yield a high value for correlation between measures where the accuracy may be meaningfully in question.

•This review, having examined the evidence on the reliability and validity of research on the site of care, by design did not address the larger question as to the clinical value of identifying a putative appropriate site of care. We cannot rule out the possibility that the clinical consequences of the manipulation treatment are to some extent site-independent.

•While it is generally recognized that using checklists to generate summary quality scores may be problematic (for example, see Whiting et al. [25,26,238]), especially when the weighting of items is arbitrary, the method which we used–employing item response theory to weight items based on the inverse of their prevalence with a set of papers, has been used previously for STARD [239] and validated for the CONSORT statement for papers within manual medicine [28].

•Systematic use of QUADAS and QAREL independently address only metrics of validity and reliability within the context of each study. It is important to bear in mind that neither characteristic necessarily implies the other. That is, an assessment may be valid but unreliable, and the obverse, reliable but not valid. The implication of either alone or both together is insufficient to define clinical utility.

•An important deficiency in the literature is the absence of significant discussion on the clinical utility of the various assessment methods.

## Conclusion

The broad search which was conducted has likely resulted in a corpus of literature which is generally representative of the current state of research in this area. Thus, where high levels of evidence were available and collective findings coherent–either favourable or unfavourable towards the use of a particular method–individual new studies are unlikely to affect the results presented herein. On the other hand, where the level of available evidence was relatively low or the findings of different studies incoherent, then new studies may well lead to new conclusions. Furthermore, the technologies themselves evolve. Thus, for example, while this review found coherent, moderate or high quality evidence which was not supportive of the use of methods that indirectly assess the tissues, technical improvements could see the future clinical validation of such methods.

Finally, while there is a need for more and better research into the underlying functional and/or pathological states that respond to manipulation, future diagnostic studies ought to focus beyond the metrics of accuracy. As posited by Bossuyt et al. [240], the value of diagnostic tests/maneuvers ought to rely on their clinical utility and capacity to change health outcomes.

## Competing interests

The authors declare that they have no competing interests.

## Authors’ contributions

JT and BB designed the study. AB and BR completed the literature searches and data extraction. JT, BB, TB, RC, BG, CG, JP and RT performed the data analysis and contributed to the writing of the manuscript. All authors read and approved the final manuscript.

## Supplementary Material

Additional file 1 QUADAS question level data. Click here for file

Additional file 2 QAREL question level data. Click here for file
